# Wild boar harbouring African swine fever virus in the demilitarized zone in South Korea, 2019

**DOI:** 10.1080/22221751.2020.1738904

**Published:** 2020-03-17

**Authors:** Seon-Hee Kim, Jisoo Kim, Kidong Son, Yongjun Choi, Hye-Sung Jeong, Yong-Kwan Kim, Jung-Eun Park, Yoon-Jee Hong, Song-I. Lee, Seung-Jun Wang, Hyun-Seo Lee, Won-Meong Kim, Weon-Hwa Jheong

**Affiliations:** Biosafety Research Team, Environmental Health Research Department, National Institute of Environmental Research, Incheon, South Korea

**Keywords:** African swine fever virus, wild boar, genotype II, demilitarized zone, South Korea

## Abstract

The African swine fever virus (ASFV) was first detected in wild boar in the Demilitarized Zone, a bordered area between South and North Korea, on 2 October 2019. Phylogenetic analyses of ASFV genes encoding p72 and CD2v indicated that the causative strain belongs to genotype II and serogroup 8, respectively, and contained additional tandem repeat sequences between the I73R and the I329L protein genes.

African swine fever (ASF) is one of the most lethal diseases among domestic pigs and wild boars, reported by OIE. ASF is a devastating haemorrhagic infectious disease characterized by severe depression, with fatality rates approaching 100% with no vaccine: it is caused by the ASF virus (ASFV; family *Asfarviridae*, genus *Asfivirus*), a large, enveloped, double-stranded DNA virus [1]. ASF was first reported in Kenya in 1921 as being endemic to sub-Saharan countries and Sardinia (Italy). After genotype II ASFV isolates were derived from Georgia and Eastern Africa [2], the virus reportedly propagated to adjacent regions in Russia, the Caucasus mountain region and numerous neighbouring Western European countries [3]. ASF was recently introduced into East Asian countries including China (2018) and continued to propagate within East and Southeast Asia (Mongolia, Vietnam, Laos and the Philippines). Furthermore, ASF was reported in Usi county, Chagang province, North Korea on 23 May 2019. The farm is in the north western region of North Korea, immediately proximal to Liaoning province, China. Since ASF has propagated throughout Asia, efforts have been initiated in South Korea to prevent ASF, including border fences, disinfection and strict quarantine measures.

Despite active preventive policies, on 2 October 2019, a dead wild boar with clinical signs like those of ASF was reported in the Demilitarized zone (DMZ) that separating South and North Korea near Yeoncheon County, Gyeonggi province, South Korea (Figure 1, panel A).

**Figure 1.**
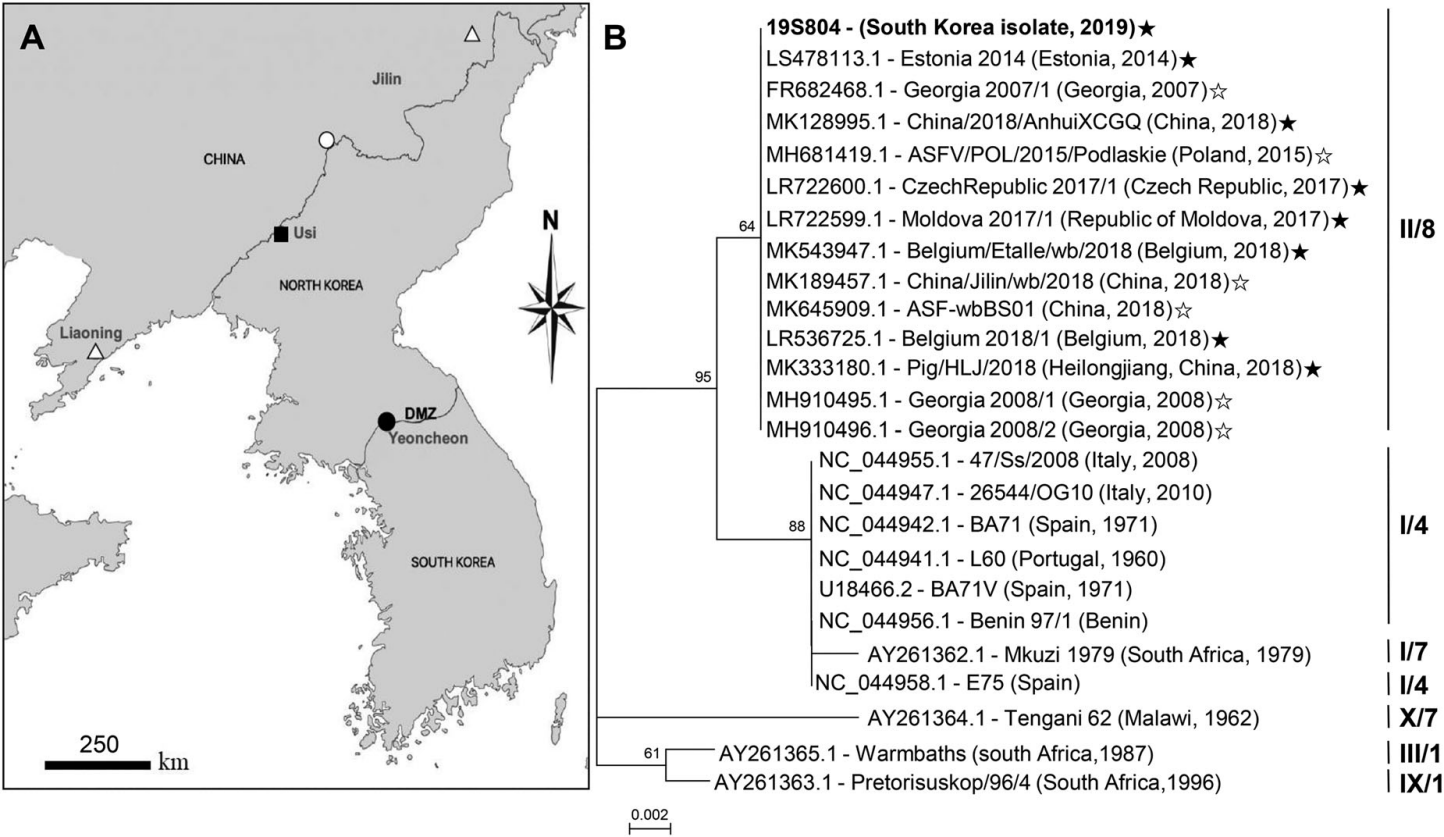
Map indicating the location and phylogenetic analysis of the causative virus strain (isolate 19S804) of an African swine fever virus outbreak in South Korea. (A) African swine fever outbreaks in South Korea in the wild boar (●), North Korea in the backyard pig (▪), China in the domestic pig (△) and China in the wild boar (○). (B) Comparison of partial fragments of the South Korean ASFV isolate 19S804 with those of other ASFV isolates. Roman numbers to the left indicate p72 genotypes. Numbers to the right indicate the CD2v serogroup. The black star at the end of the gene number indicates IGR II; white star, IGR I variant. GenBank accession numbers are provided for all sequences.

This case was reported to the National Institute of Environmental Research (NIER) and blood was sampled from the dead wild boar and sent to the Biosafety Level 3 laboratory of NIER for the confirmation of ASFV infection.

DNA was extracted from blood samples using a Maxwell RSC Blood DNA Kit (Promega, Medison, WI) and TaqMan qRT-PCR (Applied Biosystems) was performed to assess the ASFV infection in accordance with the Terrestrial Manual of the OIE [4]. The specific viral target genomic region was readily amplified via (Ct value, 20). For more accurate confirmation of the ASFV infection, conventional PCR was further performed with a PPA-1/PPA-2 primer set for *VP73* [5]. And sequenced, the first ASFV was eventually confirmed in the wild boar in South Korea (viral isolate 19S804).

Additional PCRs were performed using this DNA sample to investigate the genotype (*B646L* gene) and serotypes (*EP402R* gene) of the Korean isolate and the length of its polymorphic tandem repeat sequence (TRS). The genotype would help predict the origins of the virus and/or differentiate the viral isolate from related strains [6]. We amplified 3 partial gene fragments from: *B646L* encoding the p72 capsid protein, *EP402R* coding for the cytoplasmic domain of CD2-like protein and an intergenic region (IGR) between *I73R* and *I329*, using specific primer sets (P72-U/P72-D) for *B646L* [7], CD2-2F/CD2-2R for EP402R [8] and IGR. Three amplicons were retained the resulting sequences in GenBank (accession nos., MN817977 MN817978, MN817979).

The *B646L* sequence of the 19S804 isolate (GenBank accession no. MN817977.1), was aligned with homologous sequences retrieved from GenBank through BLAST searches. A neighbour-joining phylogenetic tree based on partial sequence alignment indicated that the present ASFV isolate is of genotype II (Figure 1, panel B) like those curated from China. The *EP402R* sequence of 19S804 was similarly used for phylogenetic analysis to predict the serotype of 19S804, reflecting patterns in the hemadsorption inhibition. Phylogenetic analysis based on the *EP402R* sequence clustered the 19S804 isolate (GenBank accession no. MN817978.1) into serogroup 8 (Figure 1, panel B).

Finally, we compared the lengths of the TRS (5′-GGAATATATA-3′) between the present viral isolate and previously reported genotype II ASFVs. The 10-bp nucleotide was repeated thrice in the corresponding genomic region of the Korean isolate (GenBank accession no. MN817979.1), similar to that in other IGR variants in group II Belgium/2018/Etalle (GenBank accession no. MH998359.1), Volgograd/2015/Russia (GenBank accession no. KY385895.1) and China 2018/1; GenBank accession no. MH735144.1 [9,10]. In contrast, this sequence was repeated twice in the genomes of Georgia 2007/1 (GenBank accession no. FR682468.1) and china/jilin/2018/boar (GenBank accession no. MK189457.1) which were collectively categorized into IGR variant group I [10].

In conclusion, the sequence of genome fragments of ASFV isolate 19S804 from Wild boar in South Korea displayed high similarity to those of recent ASFV strains from Eastern Europe and China. However, the origin of this Korean isolate remains unclear probably owing to limited sequence information obtained herein. Outbreaks of ASFV in domestic pig of South Korea were reported in 18 September 2019 on OIE. Furthermore, relationships among ASFVs curated from North and South Korea were not addressed, because no sequences of the North Korean isolate are available. Wild boar is known as one of the mediators of ASFV propagation with human behaviour. In the case of South Korea in a special situation facing the border area, more detailed mechanical relationships are necessary to reveal the dynamics. Starting with the 19S804, wild boar ASFV begins to occur in South Korea. We currently intend to determine the complete genome sequence of isolate 19S804, which would facilitate a more detailed epidemiological investigation of this isolate.
